# Improvement of bone disease by imiglucerase (Cerezyme) therapy in patients with skeletal manifestations of type 1 Gaucher disease: results of a 48-month longitudinal cohort study

**DOI:** 10.1111/j.1399-0004.2008.00978.x

**Published:** 2008-05

**Authors:** KB Sims, GM Pastores, NJ Weinreb, J Barranger, BE Rosenbloom, S Packman, P Kaplan, H Mankin, R Xavier, J Angell, MA Fitzpatrick, D Rosenthal

**Affiliations:** a Department of Neurology, Massachusetts General HospitalBoston, MA, USA; b Department of Neurology, New York UniversityNew York, NY, USA; c University Research Foundation for Lysosomal Storage Disease, Inc.Coral Springs, FL, USA; d Department of Human Genetics, University of PittsburghPittsburgh, PA, USA; e Tower Hematology Oncology Medical GroupBeverly Hills, CA, USA; f Department of Pediatric Genetics, University of California San FranciscoSan Francisco, CA, USA; g Department of Pediatrics, The Children's Hospital of PhiladelphiaPhiladelphia, PA, USA; h Department of Pediatrics, Genzyme CorporationCambridge, MA, USA

**Keywords:** bone disease, Gaucher disease, imiglucerase, skeletal manifestations

## Abstract

Sims KB, Pastores GM, Weinreb NJ, Barranger J, Rosenbloom BE, Packman S, Kaplan P, Mankin H, Xavier R, Angell J, Fitzpatrick MA, Rosenthal D. Improvement of bone disease by imiglucerase (Cerezyme) therapy in patients with skeletal manifestations of type 1 Gaucher disease: results of a 48-month longitudinal cohort study. Clin Genet 2008: 73: 430–440. © Blackwell Munksgaard, 2008

Progressive skeletal disease accounts for some of the most debilitating complications of type 1 Gaucher disease. In this 48-month, prospective, non-randomized, open-label study of the effect of enzyme replacement therapy on bone response, 33 imiglucerase-naïve patients (median age 43 years with one or more skeletal manifestations such as osteopenia, history of bone crisis, or other documented bone pathology) received imiglucerase 60 U/kg/2 weeks. Substantial improvements were observed in bone pain (BP), bone crises (BC), and bone mineral density (BMD). Improvements in BP were observed at 3 months (p < 0.001 *vs* baseline) and continued progressively throughout the study, with 39% of patients reporting pain at 48 months *vs* 73% at baseline. Eleven of the 13 patients with a pre-treatment history of BC had no recurrences. Biochemical markers for bone formation increased; markers for bone resorption decreased. Steady improvement of spine and femoral neck BMD, measured using dual-energy X-ray absorptiometry was noted. Mean Z score for spine increased from −0.72 ± 1.302 at baseline to near-normal levels (−0.09 ± 1.503) by month 48 (p = 0.042) and for femoral neck from −0.59 ± 1.352 to −0.17 ± 1.206 (p = 0.035) at month 36. This increase was sustained at 48 months. With imiglucerase treatment, patients should anticipate resolution of BC, rapid improvement in BP, increases in BMD, and decreased skeletal complications.

Gaucher disease (GD) is an autosomal recessive lysosomal storage disease resulting from deficiency of lysosomal glucosylceramide β-glucosidase. Consequently, lysosomes of the reticuloendothelial system accumulate glycolipids, creating engorged macrophages (Gaucher cells), which displace normal tissue and result in dysfunction in many organs. Clinical manifestations of the disease include anemia, thrombocytopenia, hepatosplenomegaly, and skeletal complications including bone pain (BP) and bone crisis, cortical and medullary infarctions, cortical bone thinning, medullary expansion, osteopenia, osteolysis, osteonecrosis, and pathological fractures (1). Investigative trials and extended clinical use of recombinant β-glucocerebrosidase (imiglucerase, Cerezyme®) in more than 3000 patients have shown improvement in GD manifestations such as anemia, thrombocytopenia, hepatosplenomegaly, and physical debility (2–4).

Skeletal involvement is the most frequent debilitating complication of GD and generally has a greater impact on quality of life (QOL) than the hematologic and visceral abnormalities (4–6). Skeletal disease is variable but nonetheless progressive in its course and does not resolve spontaneously. The pathophysiologic basis of the skeletal complications of type 1 GD is incompletely understood. Altered bone formation (remodeling defects and Erlenmeyer flask deformity), altered bone resorption (osteolysis, osteopenia, and osteoporosis) and focal bone lesions (cortical and medullary infarctions and pathological fractures) may be observed (1). It is likely that complex mechanisms account for the heterogeneity of Gaucher bone disease (7–9).

Recent observations have emphasized the significance of decreased bone density in the pathobiology of GD (10). In analysis of data from the International Collaborative Gaucher Group (ICGG) Gaucher Registry, osteopenia or osteoporosis, usually detected by dual-energy X-ray absorptiometry (DXA), was reported in 300/706 patients (42%) (11). Depending on the extent of loss of bone mass, osteopenia and osteoporosis in the general population are associated with an age-related 10-year fracture risk of 8% to 45% (12). Some studies have suggested that osteopenia in GD may be treatable with imiglucerase. Improvements in bone mass were noted in pediatric patients treated with imiglucerase for over 3.5 years (13). A retrospective analysis in adults using linear mixed model analysis demonstrated that bone mineral density (BMD) as measured by DXA improved with enzyme replacement therapy (ERT) in a dose–responsive manner over time (10). Furthermore, alendronate was synergistic with ERT in improving bone density and clinical outcomes (14). In retrospective studies, imiglucerase treatment markedly decreased the occurrence of bone crises (BC) and ameliorated BP (4, 15).

This study provides the first prospective, long-term analysis that extends to 4 years of treatment of the effects of ERT on DXA-measured BMD in a single cohort of primarily adult GD patients and is complementary to other studies. In previously reported analyses from the current prospective clinical trial, we have shown that imiglucerase improves health-related QOL in patients with skeletal manifestations of GD (16). Here, we confirm that imiglucerase improves the clinical consequences of bone manifestations in GD including BP, BC, and BMD measurements.

## Methods

This was a multicenter, open-label, single-cohort, prospective study using within-patient comparisons to baseline to evaluate the effectiveness of imiglucerase in treating skeletal manifestations of type 1 GD in patients who had not previously received enzyme therapy. All patients signed an informed consent form, approved by institutional review boards, prior to enrollment in the study. The study was performed in compliance with Good Clinical Practice and the Declaration of Helsinki.

Inclusion requirements included confirmed type 1 GD, age 10–70 years, and at least one of the following: history of at least one bone crisis, osteoarticular necrosis, medullary infarction, lytic lesions, pathological fractures or fractures related to GD, marrow infiltration with a unilateral Rosenthal magnetic resonance imaging (MRI) score ≥3 (7), bone density by DXA or quantitative computerized tomography scan Z score ≤−1.5, or Erlenmeyer flask deformity. The inclusion criteria were amended 12 months into the study to require DXA femoral neck T score ≤−1.0. The exclusion criteria included peri-menopausal status, major concurrent disorders, non-ambulatory status, more than one joint replacement, prior enzyme therapy (enrollment was permitted up to 12 weeks after first infusion of enzyme), gene therapy, bone marrow transplantation, and use of medications known to affect bone homeostasis, including bisphosphonates.

Patients were treated on study from September 1997 to June 2004. Patients received 60 U/kg/2 weeks of commercial imiglucerase by intravenous infusion for the first 24 months of therapy. After 24 months, the dose could be reduced to 45 or 30 U/kg/2 weeks if a central review committee determined that the patient had adequate hematological and visceral function and improvement in skeletal manifestations. Treatment duration was up to 48 months.

Medical history and physical examination, assessments of physician-reported BP and BC, complete blood counts with differential, and recording of concomitant medications, procedures, and illnesses were performed on entry and thereafter every 3 months. BP was assessed by patient report on a six-point scale: none, very mild, mild, moderate, severe, or extreme. Bone crisis was defined as pain with acute onset requiring immobilization of the affected area, narcotics for pain relief and may be accompanied by one or more of the following: periosteal elevation, elevated white blood cells, fever, or debilitation of >3 days.

Serum chemistries, angiotensin-converting enzyme, and serum and urine biochemical markers of bone metabolism (osteocalcin, bone-specific alkaline phosphatase, type 1 collagen crosslinked N-telopeptide, and deoxypyridinoline crosslinks) were assessed every 6 months. Bone biochemical marker assays were performed in the laboratory of Thomas Clemens at the University of Cincinnati College of Medicine. Liver and spleen volumes were measured every 12 months using MRI.

To ensure consistency of results, DXA scans of the lumbar spine, femur, and forearm were performed for all patients at the same site (Massachusetts General Hospital) with a Hologic 4500QDR. The calibration of the scanners as well as reporting of spinal density T scores and Z scores were performed according to manufacturers' recommendations following standard clinical procedures. Similar procedures were followed for the femur, greater trochanter, and the forearm (1/3 radius + ulna). We chose to focus primarily on Z scores in presenting BMD changes for this study population given that the patients were not a homogeneous group in terms of age and gender. Evaluation of treatment-induced changes in T scores over a period of years might be confounded by expected age-related changes during that period. Additional skeletal evaluations included single-energy quantitative computerized tomography, MRI quantitative chemical shift imaging, X-ray assessments, and coronal MRI. These modalities were used to evaluate skeletal manifestations as reported in Table 3. Detailed results from these analyses will be reported separately.

**Table 3:** Skeletal events

Skeletal manifestation	Patients with evidence, *n* (%)		Occurrence of post-baseline events (month)			
		
	At baseline or by history	Post-baseline	0–12	>12–24	>24–36	>36–48
Medullary infarction	12 (36)	4 (12)	2	2	0	0
Osteoarticular necrosis	2 (6)	5 (15)	4	1	0	0
Lytic lesions	12 (36)	3 (9)	1	2	0	0
Fractures						
Long bone	1 (3)	0	0	0	0	0
Spinal	1 (3)	3 (9)	0	1	1	1
Bone crisis	13 (39)^a^	3 (9)	2	1	0	1

a Thirteen patients had a history of bone crisis at any time prior to baseline; at baseline, five of these patients reported having a bone crisis in the past 2 months.

All statistical comparisons were carried out as 2-sided tests. Analyses were conducted for the intent-to-treat (ITT) population, which included all patients enrolled in the study.

## Results

### Baseline characteristics

**Fig. 1:**
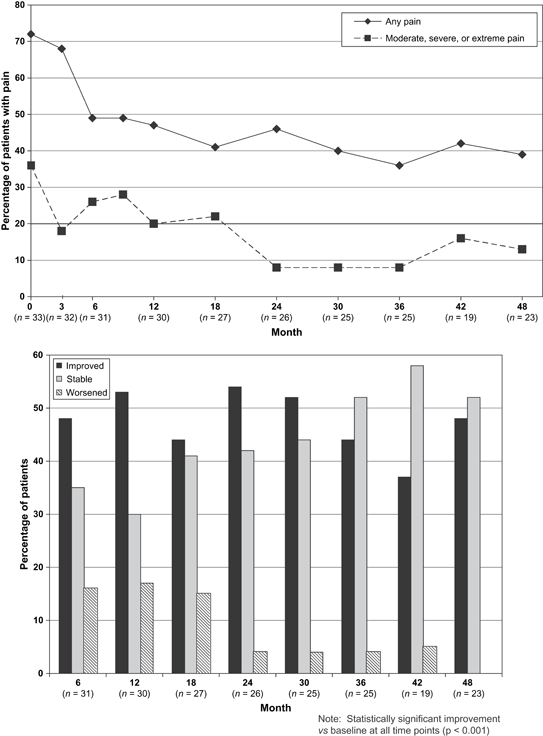
Bone pain (BP) over time. Top – self-reported pain levels over time. Patients were asked to report their BP levels as none, very mild, mild, moderate, severe, or extreme every 3 months. The percentage of patients with any pain (top line) or moderate, severe, or extreme pain (bottom line) was determined and graphed over time. At baseline, 9 patients (27%) reported no pain, 12 (36%) very mild or mild pain, 8 (24%) moderate pain, and 4 (12%) severe or extreme pain. Bottom – changes in patient-reported pain. The percentage of patients with improved, stable, or worsened pain compared with the pain level reported at baseline is shown.

Thirty-three patients were treated (Table 1). All but one patient had at least one N370S allele; 67% were N370S homozygous. A total of 23 patients (70%) were of Ashkenazic Jewish ethnicity. Only one patient was less than 22 years of age. Signs of significant pre-treatment, non-skeletal systemic disease in these patients, including splenomegaly and hepatomegaly, are listed in Table 2. Five patients (15%) had undergone total splenectomy. At baseline, 9 patients (27%) reported no skeletal pain and 24 patients (73%) reported pain – 12 patients (36%) very mild or mild pain, 8 (24%) moderate pain, and 4 (12%) severe or extreme pain (Fig. 1, top). Twelve patients (36%) had evidence of prior medullary infarction, 12 (36%) had lytic lesions, 2 (6%) had osteoarticular necrosis, 1 (3%) had long bone fracture, and 1 (3%) had spine fracture (Table 3). For the lumbar spine, 18/31 patients with baseline data (58%) were osteopenic at baseline by DXA T score (T score ≤−1.0) and 6 patients (19%) had osteoporosis (T score ≤−2.5). For the femoral neck, 26/32 patients (81%) were osteopenic at baseline. None of the enrollees had a history of joint replacement.

**Table 1:** Patient disposition and demographics

Parameter	Measure
Number of patients treated	33
Number of patients not completing study, *n* (%)	10 (30)
Principal reason for withdrawal, *n* (%)	
Adverse events	1 (3)
Uncooperative (could not make visits)	1 (3)
Wishes to withdraw	3 (9)
Sponsor ended study	3 (9)
Others (lung cancer and circumstances prevented further participation)	2 (6)
Study treatment duration, months, median (range)	49.4 (4.2–58.6)
Time of last efficacy visit, *n* (%)	
≤12 months	6 (18)
>12 to ≤24 months	0
>24 to ≤36 months	4 (12)
>36 to 48 months	23 (70)
Gender, *n* (%)	
Male	19 (58)
Female	14 (42)
Ethnicity, *n* (%)	
Ashkenazi Jewish	23 (70)
Non-Jewish Caucasian	6 (18)
African-American/Caribbean	1 (3)
Hispanic	2 (6)
American Indian	1 (3)
Others	2 (6)
Genotype, *n* (%)	
N370S N370S	22 (67)
N370S-L444P	6 (18)
N370S-D409H	1 (3)
N370S-IVS2+1	1 (3)
N370S-unknown	2 (6)
Unknown	1 (3)
Patients with total splenectomy, *n* (%)	5 (15)
Median age at Gaucher symptom onset, years (range)	32.0 (11.0–52.0)
Median age at consent, years (range)	43.0 (12.0–70.0)
Median weight at baseline, kg (range)	64.2 (34.0–105.0)
Median height at baseline, cm (range)	169.9 (146.5–190.5)

**Table 2:** Non-skeletal measures of disease

Measure	Baseline result	Month 48 result^a^
Platelet count, ×10^9^/l, mean (SD)	*n* = 33, 120 (60.7)	*n* = 23, 198 (59.6)
Hemoglobin, g/dl, mean (SD)	*n* = 33, 12.9 (1.48)	*n* = 23, 14.5 (1.72)
Liver size, ×normal, mean (SD)	*n* = 29, 1.4 (0.52)	*n* = 22, 1.0 (0.24)
Spleen size, ×normal, mean (SD)	*n* = 26, 6.5 (3.34)	*n* = 19, 3.6 (1.74)

a p Value <0.001 testing the hypothesis that the mean percent change is equal to zero using a *t*-test.

### Study compliance and dosing

Of 33 treated patients, 27 patients (82%) had efficacy evaluations at 24 months; 23 patients (70%) completed the 48-month study and 10 patients did not complete the study (Table 1). Most patients received an imiglucerase dose of 60 U/kg/2 weeks for the entire study. Nine patients had dose reductions from 60 to 45 or 30 U/kg generally after the month 24 visit (one patient had dose reduction to 45 U/kg at month 18). The median dose at all time points was 60 U/kg/2 weeks, except for month 45 where it was 56.1 U/kg/2 weeks. Missed infusions were uncommon. Twenty-three patients (70%) missed no infusions, and only two patients missed five or more infusions.

### Bone pain

Reduction in patient-reported pain levels was evident by month 3 (Fig. 1, top). By month 6, the number of patients with any reported BP had fallen from 24/33 (73%) to 15/31 (48%). In months 18 through 48, the percentage of patients with any pain plateaued at approximately 40%. Twelve of 33 patients (36%) reported moderate, severe, or extreme pain at baseline; only 6/32 patients (19%) reported these pain levels at month 3, and less than 20% of patients reported these levels at any time point from month 24 onward. At month 48 (*n* = 23 completing patients), 14 patients (61%) had no pain, 6 patients (26%) had very mild or mild pain, 3 patients (13%) had moderate pain, and no patient had severe or extreme pain. These changes were not because of withdrawal of patients with pain from the study – of the 10 patients who discontinued, only 3 had any pain at their last evaluation (1 very mild, 1 mild, and 1 severe). Pain reported by the 23 patients who completed the study was similar to reports given by the total population of 33 patients (data not shown).

**Fig. 2:**
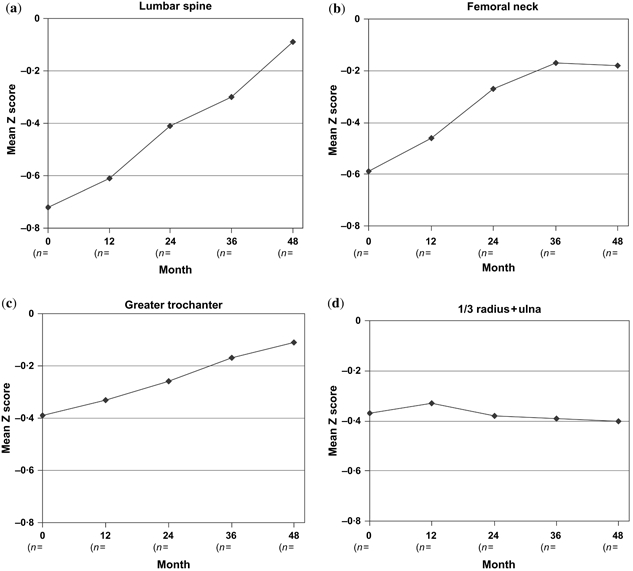
Bone density (mean dual-energy X-ray absorptiometry Z scores, reflective of age-adjusted normal) as measured in the spine (**a**), femoral neck (**b**), greater trochanter (**c**), and 1/3 radius + ulna (**d**). P values test the hypothesis that the mean change is equal to zero (*t*-test). *p < 0.05.

Patient reports of BP over time were compared with their reported pain at baseline for improvement, worsening, or no change (Fig. 2, bottom). Patients with pain improvement over baseline were noted at all time points. In addition, the percentage of patients with worsening BP compared with baseline decreased over time to ≤10% by month 24. These improvements were statistically significant (p < 0.001 at all time points). Of the nine patients with no pain at baseline, only three reported BP at any time during the course of the study.

### Bone crisis and skeletal complications

The number of patients reporting BC decreased in the first 12 months and remained below baseline for the remainder of the study (Table 3). At study entry, 13/33 patients (39%) had a history of BC, with 5 of these patients (15%) reporting BC in the 2 months immediately preceding initiation of imiglucerase. In the first year of the study, only two patients reported new BC (*n* = 32 at month 12). After month 12, only one patient reported a bone crisis per 12-month period, and between 24 and 36 months, no crises were reported (*n* = 29, 27, and 23 patients with evaluations at months 24, 36, and 48, respectively). Overall, three patients reported BC post-baseline; two of these patients had prior history of bone crisis. Eleven of 13 patients with a history of BC (85%) did not have a crisis during the 48-month study.

Most incidents of medullary infarctions, osteoarticular necrosis, the appearance of lytic lesions, and/or long bone and spinal fractures occurred within the first 24 months of the study; only two events, both spinal fractures, were noted past month 24 of the study (Table 3). Events during the study were much more likely to occur in patients with pre-existent skeletal complications. All post-treatment complications were identified in the course of radiological monitoring and were not associated with clinical symptoms.

### DXA bone density measurements

Mean lumbar and femoral neck DXA Z scores (Fig. 2) and T scores (data not shown) improved progressively with imiglucerase treatment. In the lumbar vertebrae, the mean baseline Z score ± SD was −0.72 ± 1.302, which corresponds to a mean density of 0.926 ± 0.142 g/cm^2^ and a mean T score of −1.27 ± 1.257. A trend toward improvement in DXA Z score of the lumbar vertebrae L1 to L4 over baseline was apparent by month 12, while statistically significant improvement was observed starting at month 24 and continuing through month 48 (p < 0.05). By month 48, the mean Z score had reached near-normal levels (−0.09 ± 1.503, corresponding to a mean density of 1.003 ± 0.183 g/cm^2^ and a mean T score of −0.67 ± 1.587). Improvements were also seen in the femoral neck. Mean Z scores increased from −0.59 ± 1.352 at baseline (corresponding to a mean bone density of 0.769 ± 0.162 g/cm^2^ and a mean T score of −1.53 ± 1.274) to −0.17 ± 1.206 at month 36 (p = 0.035, corresponding to a mean bone density of 0.791 ± 0.175 g/cm^2^ and a mean T score of −0.88 ± 1.244). This level of change was sustained at 48 months, but because of the loss of two patients, statistical significance was lost (p = 0.231). However, significant changes did not occur either at the greater trochanter or in the distal third of the radius and ulna.

A summary of DXA results and skeletal events for individual patients is shown in Table 4. Improvements in BMD were not dependent on splenectomy status, age, baseline BMD, or occurrence of spinal fractures during the study (data not shown).

**Table 4:** Summary of patient results

Patient	Sex, age at consent (years)	Splenectomy?	Genotype	Baseline spine DXA Z score	End of study spine DXA Z score	Baseline femur DXA Z score	End of study femur DXA Z score	Last DXA time point (months)	Skeletal events^a^	
									
									Baseline	After
5001	F, 40	N	N370S/N370S	−0.09	−0.10	−1.62	−1.10	12	L	
5002	F, 51	Y	?/?	−0.42	−0.40	−0.96	−0.50	48	B	B
5601	M, 32	N	N370S/N370S	−1.29	−1.50	−1.50	−0.80	48		
5602	M, 22	N	N370S/N370S	−0.60	0.00	0.72	0.90	48	L	OBLM
5603	M, 53	N	N370S/N370S	3.30^b^	2.40	1.43	1.20	48		
5604	M, 59	N	N370S/N370S	−1.81	−0.90	−1.75	−1.40	48	OB	S
5605	M, 36	N	N370S/N370S	−0.66	0.00	−0.76	0.00	48		
5606	M, 22	N	N370S/ISV2+1	0.31	3.90	0.61	−0.30	48	BLM	OLMS
5607	F, 12	N	N370S/N370S	−1.00	−0.20			48	BLM	
5608	M, 37	Y	N370S/N370S	−0.36	0.30	−0.88	−0.10	48		
5609	M, 42	N	N370S/N370S	−3.20	−3.30	−1.30	−1.20	24	BLM	OM
5701^c^,^d^	F, 51	N	N370S/N370S	−2.06	−1.30	−2.19	−1.40	48	LM	
5702	M, 38	N	N370S/L444P	−1.47	−0.80	0.31	0.60	48	LM	
5703	F, 28	N	N370S/D409H	−0.90	−0.50	−1.80	−1.70	48		
5704^c^	M, 55	N	N370S/N370S	−2.50	−1.20	−0.80	−0.30	36	LM	L
5801	F, 40	N	N370S/?	−0.35	0.05	−1.10	−1.22	12	BLM	BO
6001	M, 68	N	N370S/?	1.30	1.90	3.80	2.90	48	OBLM	LM
6002	M, 64	N	N370S/N370S	−0.04	0.00	0.02	−0.20	36		
6201^d^	F, 44	Y	N370S/L444P	−2.05	−2.20	−1.75	−1.70	48	B	
6202	F, 54	N	N370S/N370S	0.76	0.90	−0.49	0.00	48	B	
6203^d^	F, 70	N	N370S/N370S	−0.58	−0.30	−0.68	−0.40	48		L
6204	M, 38	N	N370S/N370S	−1.45		−2.39		BL	B	
6205	F, 48	N	N370S/N370S	−0.16	−0.40	−0.54	−0.50	48	M	S
6206	F, 39	Y	N370S/L444P	−2.40	−1.80	−1.70	−1.50	36		
6301	M, 41	N	N370S/N370S	−1.39	−0.60	−0.79	−0.20	48		
6302	M, 44	N	N370S/N370S	−1.70	−1.60	−1.07	−0.50	48		
6303	F, 56	N	N370S/N370S	−0.64		−1.66		BL	LS	
6304	M, 64	Y	N370S/N370S	3.38	2.60	0.54	0.50	48		
6305	M, 54	N	N370S/L444P	−0.45	−1.40	−0.88	−1.80	48		O
6306^d^	F, 61	N	N370S/L444P	1.52	1.52	−0.62	−0.47	12	BLM	
6307^e^	M, 34	N	N370S/N370S							
6308	F, 38	N	N370S/L444P	−0.96	−0.80	−1.03	−1.00	48	BM	
6309	M, 43	N	N370S/N370S	−1.13	−1.10	2.57	2.20	48	BML	

DXA, dual-energy X-ray absorptiometry; ?/?, unknown/unknown allele mutations.

a B, bone crisis; L, lytic lesion; M, medullary infarction; O, osteoarticular necrosis; S, spinal fracture.

b No baseline data available. 3.30 is at 24 months.

c Patient received bisphosphonates (protocol deviation). Patient 5701 received pamidronate from month 21 and patient 5704 received fosamax from month 18.

d Patient received hormone replacement therapy.

e Patient 6307 discontinued from the study after one infusion and no baseline scans because of inability to comply with visit schedule.

An additional analysis was performed excluding patients from the ITT population for factors that might have affected the analysis of BMD [patients 5603, 6204, 6303, and 6307 were missing baseline or follow-up data; patient 5606 was excluded from the lumbar vertebrae analysis because of a compression fracture; patient 5607 was the only non-adult (12 years old); patients 5701 and 5704 were excluded for bisphosphonate use and patients 6201 and 6203 for hormone replacement therapy use]. In this analysis, changes in the lumbar vertebrae were statistically significant at months 24 (p = 0.046) and 36 (p = 0.011), although the magnitude of changes were smaller than in the overall population (mean Z scores increased from −0.54 ± 1.346 at baseline to −0.25 ± 1.151 at month 48, corresponding to a mean bone density of 0.0954 ± 0.130 g/cm^2^ and a mean T score of −1.00 ± 1.160 at baseline *vs* a mean bone density of 1.000 ± 0.116 g/cm^2^ and a mean T score of −0.75 ± 1.019 at month 48). Statistically significant changes in the femoral neck Z score were observed at month 36 (p = 0.044), and the magnitude of changes were similar to the overall population (mean Z scores increased from −0.48 ± 1.342 at baseline to −0.09 ± 1.216 at month 48, corresponding to a mean bone density of 0.788 ± 0.166 g/cm^2^ and a mean T score of −1.36 ± 1.280 at baseline *vs* a mean bone density of 0.813 ± 0.166 g/cm^2^ and a mean T score of −0.79 ± 1.192 at month 48).

### Markers of bone metabolism

**Fig. 3:**
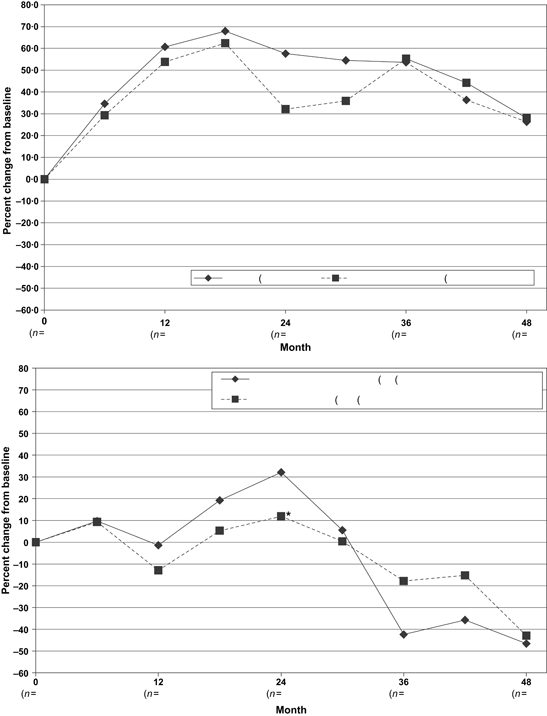
Percent change from baseline in median biomarker values of intent-to-treat population. Top, markers of bone formation. *p < 0.05; **p < 0.001. Bottom, markers of bone resorption. *p < 0.05.

Although median baseline measurements of osteocalcin, bone-specific alkaline phosphatase, N-telopeptide crosslinks, and deoxypyridinoline (D-PYD) at baseline fell within the normal range, median post-treatment measurements for bone formation markers, osteocalcin and bone-specific alkaline phosphatase increased by approximately 60% in the first 12 months of the study and consistently remained above baseline values throughout the study (Fig. 3, top). These results reflected statistically significant changes from baseline at all time points. At month 12, 25 patients (93%) had an increase in osteocalcin over baseline and 24 patients (89%) had an increase in alkaline phosphatase over baseline; at all time points, at least 74% of patients showed increases in these markers over baseline, although some increases were small. Between 36 and 48 months, median levels of bone resorption markers, N-telopeptide crosslinks and D-PYD decreased approximately 20–40%, although these changes were generally not statistically significant (Fig. 3, bottom). For each of these measures, at month 36, 15 patients (68%) had a decrease from baseline. These results suggest a shift favoring new bone formation relative to bone resorption and are consistent with the observed progressive increase in vertebral and femoral neck BMD.

### Hematologic and visceral responses and safety

Improvements in platelet count, hemoglobin, and spleen and liver volumes were consistent with other studies of imiglucerase in patients with GD (4) (Table 2). The safety results from this study were consistent with the results expected based on the package labeling and results from previous studies (4). Overall, the most common adverse events were chills (seven events), flushing (six events), and arthralgia (six events), each reported in four patients (12%). One patient withdrew from the study and treatment because of a severe infusion reaction, including anxiety, chest pain, hypertonia, chills, tachycardia, and vomiting, from which he recovered without sequelae.

## Discussion

Our study results show a decrease in BP and skeletal complications and an increase in BMD in patients with type 1 GD treated with imiglucerase. Despite the predominance of the N370S genotype in these patients and the relatively mild hematological and visceral disease in this cohort, the patients had evidence of serious skeletal disease at baseline. When this study was initiated, imiglucerase was the sole proven effective therapy available for the hematological and visceral manifestations of type 1 GD found in our patients. Therefore, there was neither any alternative treatment arm proposed nor was a placebo arm included. There is, in fact, no evidence suggesting that established untreated Gaucher bone disease is spontaneously reversible (1). Untreated, BP and bone crisis are frequently associated with irreversible bone pathology that leads to morbidity, deformity, fractures, multiple orthopedic surgeries, decreased QOL, and early mortality.

Decreased BMD is commonly observed in both men and women with type 1 GD (17). Several studies have assessed the effect of ERT on BMD in GD, with variable results. Rosenthal et al. observed significant increases in cortical thickness after 42 months of treatment (13). Using DXA, Ciana et al. reported statistically significant improvements in BMD at a mean follow-up time of 4.5 years (18). However, Schiffmann et al. found a decrease in BMD using QCT of the lumbar spine in patients receiving ERT (19) and Lebel et al. reported a mixture of improvement, no change, or decline over a period ranging from 24 to 108 months using DXA (20). These results are difficult to compare and interpret because the studies were performed in different patient populations (pediatric *vs* adult patients and different doses of ERT) and different methodologies were used to assess BMD.

DXA measurements are quantitative, reproducible, widely available in developed countries, and relatively inexpensive. The progressive increase in lumbar spine and femoral neck BMD that we observed supports previous suggestions that serial DXA measurements of these sites be included when monitoring the response to treatment in patients with type 1 GD (4) and that improving lumbar and femoral BMD is an appropriate therapeutic goal (21). The increases in BMD noted in this paper, which appeared more quickly than noted in some other studies using various doses of imiglucerase in GD (18, 20), suggest that the dosage used in this study (60 U/kg/2 weeks) may be an appropriate starting dose in osteopenic patients. Additionally, the lack of change in forearm BMD measurements indicates that assessment of this site may not provide clinically meaningful information when used in the monitoring of patients with type 1 GD.

Our results are consistent with, and complementary to, the recently published retrospective analysis from the ICGG Gaucher Registry. This analysis, in 342 ERT-treated patients, showed that pre-treatment lumbar DXA Z scores were significantly below normal but progressively improved with treatment over an 8-year period with a significant dose–response relationship (10). In our study, patients received imiglucerase 60 U/kg every 2 weeks. This dose was noted to be the most effective in the Gaucher Registry study. Unlike the Gaucher Registry analysis, however, our trial is not confounded by possible concurrent use of bisphosphonates or by variance in DXA machine type or protocol, although other potential confounders such as smoking and use of female hormone replacement started prior to ERT are not excluded. In our patient cohort, improved BMD occurred concurrently with decreases in BC and BP. Of note, the incidence of fractures was low throughout the 48 months of the study. Few events occurred after month 24, and it is possible that events occurring early in the study were because of diseases present at baseline. Our study, however, neither demonstrated a definitive correlation between BMD and skeletal complications nor do we have any evidence as yet that increasing BMD in patients with type 1 GD will result in long-term reduction in fracture risk as has been shown in women with post-menopausal osteoporosis (22). Nevertheless, improving BMD appears to be of benefit in other chronic inflammatory diseases with associated osteopenia such as rheumatoid arthritis and Crohn's disease (23, 24), and it is likely that further cumulative experience with ERT in GD will show similar results. The treatment-associated decreases in BP, and in occurrence of BC observed in this prospective study, are consistent with the retrospective experience reporting improvement in BP and BC in larger number of patients from the International Gaucher Registry (15).

Although some previous studies have found that classical biochemical markers for bone formation (osteocalcin and bone-specific alkaline phosphatase) are of limited clinical value for monitoring the response to ERT in patients with type 1 GD (18), our results, which demonstrate a consistent increase in these markers, suggest that further investigation of the utility of these markers to monitor bone formation is warranted. Increases in bone formation markers from baseline values have been used as an index of response in patients treated for post-menopausal osteopenia (25). Adjuvant treatment of osteopenia/osteoporosis in patients with type 1 GD has largely focused on inhibition of osteoclasts and retardation of bone resorption with bisphosphonates (14). However, the lack of consistent changes in markers of bone resorption (N-telopeptide crosslinks and D-PYD) suggests that they may be of limited utility in monitoring bone response to ERT in GD. Our preliminary findings revealed that imiglucerase may enhance bone formation in osteopenic GD patients and confirm the results of Schiffmann et al. (19). Prospective trials using anabolic bone agents may be worthwhile to undertake in imiglucerase-treated GD patients with persistent osteopenia to determine whether bone formation and BMD can be further enhanced.

In summary, this prospective study confirms that ERT with imiglucerase improves the major symptomatic manifestations of Gaucher skeletal disease, bone crisis and BP, decreases the risk of skeletal events (infarction, lytic lesions, and fracture), and increases lumbar spine and femoral neck BMD during the first 4 years of treatment. Our results suggest that early initiation of treatment in symptomatic patients can substantially alleviate discomfort and may prevent potentially disabling bone complications and overall morbidity.
